# COVID-19: a perspective for lifting lockdown in Zimbabwe

**DOI:** 10.11604/pamj.2020.35.2.23059

**Published:** 2020-04-30

**Authors:** Mathias Dzobo, Itai Chitungo, Tafadzwa Dzinamarira

**Affiliations:** 1 Department of Medical Laboratory Sciences, University of Zimbabwe, Harare, Zimbabwe; 2 Department of Public Health Medicine, School of Nursing and Public Health, University of KwaZulu-Natal, Durban, 4001, South Africa

**Keywords:** COVID-19, lockdown, Zimbabwe

## Abstract

The Coronavirus disease 2019 (Covid-19) scourge has challenged the world’s health systems and presented multiple socio-economic and public health challenges to the states it has affected. Zimbabwe has been affected by the pandemic, and in response, the government has set up an array of measures, including a national lockdown, to curb transmission. While it is critical to maintain such vigorous containment measures, socio-economic pressures in Zimbabwe will challenge the sustainability of the lockdown. Given the potential for lift of the lockdown before the Covid-19 pandemic ends, we discuss the Covid-19 pandemic situation in Zimbabwe and viewpoints on important considerations and strategies for lifting the lockdown.

## To the editors of Pan African Medical Journal

Coronavirus disease 2019 (Covid-19) is an infectious disease caused by the severe acute respiratory syndrome coronavirus 2 (SARS-CoV-2) virus [1]. The outbreak, which was originally identified in Wuhan, China in December 2019 [2], was declared to be a Public Health Emergency of International Concern by the World Health Organization (WHO) on 30 January 2020 [3] and as a pandemic on 11 March 2020 [4]. The SARS-CoV-2 virus has infected more than 2.2 million people, with over 140,000 fatalities in 210 countries as of 16 April 2020 [5]. The pandemic has resulted in the largest-ever amount of shutdowns/lockdowns worldwide, with more than half of humanity under lockdown as of 3 April 2020 [6]. To date, social distancing and proper hygiene have been reported as the best defense against this pandemic [5]. In an effort to enforce social distancing, most African countries have implemented lockdown orders. Lockdowns have been shown to be beneficial in combatting Covid-19 in Wuhan, China [7], the former epicenter of the pandemic. In Italy, after an initial laissez-faire approach to the pandemic, a lockdown was implemented 39 days after the first case was identified, and the number of new cases began to fall at Day 13 of the confinement [8-10]. Most African countries´ economies cannot sustain being shut down for extended periods. Zimbabwe is one of the countries faced with this dilemma. In this perspective article, we give a brief description of the Covid-19 situation in Zimbabwe and discuss viewpoints on important considerations and strategies for lifting the lockdown.

**The COVID-19 situation in Zimbabwe:** the government of Zimbabwe declared Covid-19 a national disaster on 17 March 2020 [11]. This allowed the state to mobilize resources for preparedness and probable containment efforts. In the early days, there were concerns of poor testing capacity impeding the identification of cases among travelers returning from high-risk countries [12]. The first case to be detected was on 21 March 2020. In the space of a few days, multiple measures to curb the SARS-CoV-2 virus were put in place. These included a ban on all public gatherings, including church services, weddings, and all international sporting fixtures. Multiple hand-washing basins were installed around the country. The government also ordered the closure of schools and designated three hospitals as quarantine facilities for Covid-19. On 30 March, the country went into national lockdown for an initial period of 21 days. At the time of writing of this article, the lockdown had been extended for a further 14 days. As of 21 April 2020, there were 25 laboratory-confirmed cases in Zimbabwe, with 3 deaths. The government is now considering strategies to lift the lockdown given the current sporadic cases and the potential for a new cycle of Covid-19 outbreak if containment measures are not in place.

**The decision to lift the lockdown:** China was the first country to lift the lockdown after almost 100 days of lockdown in Wuhan. The decision was largely influenced by the successes of strict social distancing, massive testing, and contact tracing that helped reduce transmission and the numbers of new cases [13]. The WHO released a list of six criteria that countries need to consider before lifting the lockdown [14]. With respect to the WHO criteria, Zimbabwe is yet to satisfy some of the requirements, particularly issues surrounding diagnostic testing, isolation, and contact tracing. However, from economic and social points of view, confinement measures are not sustainable in the long run. The livelihoods of the majority of Zimbabweans depend on the informal sector, and further delay in lifting the lockdown would mean hunger for most households. There are fears that people may die from non-Covid-19-related deaths if the national economy goes into a slump due to the burden of the pandemic. Already, there is an active malaria outbreak in the northeastern parts of the country [15]. Zimbabwe has to come up with a responsible lockdown exit framework that is suited to the socioeconomic structure of the country. This framework has to address the need for increased awareness, surveillance, and diagnostic testing whilst making efforts to restore the social lives of citizens. This does not come without its challenges, mostly funding and resistance from the population. This paper proposes a framework that the Government can adopt post-lockdown to curb the spread of the pandemic.

**Awareness and education:** the global spread of the Covid-19 pandemic means that it will be a long time before the pandemic can be eliminated, and the fear of resurgences is a reality. With no vaccine or effective antiviral drug likely to be available soon [16], Zimbabwe would have to remain on high alert and expect the worst. A vigorous campaign of awareness and education must be adopted across the country. According to the WHO criteria for lifting lockdowns [14], communities should be educated, engaged, and empowered to adjust to a “new norm”. The government has so far acted to raise awareness through state-run media by running adverts, producing jingles, and printing posters. This media campaign has to continue. The national Covid-19 hotline (2-0-1-9) will have to remain active to enable easy reporting of cases from all corners of the country. The communication infrastructure has to be maintained, even post-Covid-19, to enable effective response in the event of another outbreak or health emergencies. In West Africa, communication and awareness infrastructure initiated during the 2013-2016 Ebola outbreak was leveraged to respond immediately to the current pandemic [17].

**Laboratory capacity:** a notable weakness in Zimbabwe´s response to the Covid-19 pandemic has been the low number of tests carried out on the population. Among the WHO criteria that countries need to consider before lifting lockdowns is the ability of health systems to quickly detect, test, isolate, and treat new cases, as well as to trace close contacts. The Ministry of Health and Child Care (MoHCC) has targeted an ambitious 33,000 tests per month, but as at 20 April, the government had conducted a total of 3308 tests [18]. The government could take a leaf from South Africa´s book: Zimbabwe´s southern neighbor took advantage of the lockdown to roll out massive testing and household screening. As of 20 April, South Africa had conducted 121,510 tests [19]. South Africa´s handling of the pandemic has earned it global admiration. The number of cases there has declined in the last two weeks, and the actual situation in South Africa has shown a different trajectory from the expected exponential infection increase. South Africa has shown that exponential spread can be avoided by upscaling testing and engaging in massive screening and contact tracing. Zimbabwe has to engage in massive testing in order to gather as much data as possible that can be used to infer patterns of spread.

Currently, the only positive cases have been reported in 4 of the country´s 10 provinces [18]. As the government considers life after lockdown, it has to expand diagnostic testing as this has the advantages of picking out mild and asymptomatic cases of Covid-19, which are estimated to comprise around 80% [20]. Such cases have been blamed for contributing to the increased community spread of the virus [21]. Widespread Covid-19 testing will likely generate large amounts of epidemiological data that the government can use to assess risks and predict disease patterns in Zimbabwe. As it stands, the government cannot make such deductions based on the limited data it has on the pandemic in Zimbabwe. To upscale Covid-19 testing, more laboratory personnel will have to be hired and trained to carry out diagnostic testing. More laboratories around the country will have to be capacitated to roll out testing on a large scale [12]. The increased capacity will improve local preparedness if the Covid-19 pandemic is to hit Africa hard, as experts in public health have predicted [22]. Whether such predictions are mere speculations, Zimbabwe cannot afford to take chances, and some degree of preparedness has to characterize our healthcare system. Research institutions in Zimbabwe have been conspicuous in their absence in this pandemic. With every country making strides to come up with home-grown solutions, the government must capacitate local research through funding to promote innovations in diagnostics and therapeutic products.

**Social distancing:** without satisfying the WHO criteria for lifting lockdowns, Zimbabwe will have to move out of confinement and recover from economic losses, as well as mend social aspects of the population brought to a halt by the Covid-19-induced lockdown. One criterion is to make sure that transmission rates have reduced. Zimbabwe is not able to determine this due to the low number of tests conducted so far. Before the lockdown is lifted, the government will need to come up with measures of preventing the spread and importation of Covid-19. Zimbabwe, like other low- and middle-income countries, faces the risk of a surge in Covid-19 infections and deaths as unrestricted movement starts to take place. The country can perhaps adopt to some extent the Chinese model of lifting the lockdown: China continued to enforce strict social distancing coupled with testing and screening in Wuhan, even after lockdown was lifted, to avoid a resurgence of infections [23]. Unlike China, Zimbabwe has a largely informal economy which lacks regulation, thereby making coordination difficult.

Our proposal to the government is to lift the lockdown in phases whilst carefully monitoring the behavior and responses of the population. The essential services like transport, the food industry, healthcare, and security forces have to be screened first to minimize local spread. This is particularly important for healthcare workers to avoid future outbreaks of nosocomial Covid-19 infections in hospital wards. Considering that people already have underlying conditions on admission to hospitals, fatalities may occur. New infection control policies specifically addressing Covid-19 should be a requirement for every company wanting to reopen after the lockdown. Employers will have to provide disinfection materials, masks, and temperature screening at single entry points. Employers will need to have small subcommittees for reporting suspected cases amongst their employees. The government will have to restrict unnecessary travel by mounting roadblocks on major highways. To prevent intercity spread of infection, it may be necessary to have travelers apply for a travelers´ pass from the responsible government unit or department. Perhaps a worrying issue is whether schools should reopen. In our view, schools can reopen on the condition that there are infection control measures put in place in schools. If schools are going to open, each school needs to have a committee responsible for the awareness and reporting of any suspected cases of Covid-19. As we approach winter, which is generally a common season for upper respiratory infections, extreme care has to be taken to mimimize spread. The teaching staff would have to be screened before schools reopen. Coming now to social gatherings, we suggest that the government delay lifting the ban on church gatherings and sporting events, while funerals may continue under the current lockdown framework of not having more than 50 people at one funeral. The wearing of masks when people are out of their homes should be enforced. Surveillance and screening must be enforced at borders and airports around the whole country to avoid importation of the virus.

**Welfare of vulnerable groups:** going into the recently extended lockdown, there have been growing calls for the government to address the plight of many Zimbabweans who risk dying of malnutrition and starvation instead of Covid-19. The lockdown has deprived many urban dwellers of their source of livelihood, which is mostly in the informal sector. To worsen things, the prices of basic commodities have increased over the lockdown period, further eroding the buying power of the majority of Zimbabweans. The government has to come up with stimulation packages and incentives for suppliers so that they can continue supplying goods and services without hiking prices. Currently, the government is implementing a programme of cushioning 450,000 people through a 90 million RTGS Dollars facility. It is a good start, but divided equally, it means that each person will get 200 RTGS dollars, which is worth six loaves of bread at current pricing. In a country of approximately 16 million people, the state has to expand the programme to benefit more deserving people. Zimbabwe´s health sector was already overburdened before the Covid-19 pandemic by communicable and noncommunicable diseases like tuberculosis (TB), HIV, and diabetes and the current active malaria outbreak in Uzumba district. The government has to cater for these patients and ensure the availability of medical care and treatment. A plan for managing these deficits should be put in place as the country heads into the post-lockdown period. The government has to be sensitive to the need to reestablish social organization, while at the same time ensuring the safety and health of its citizens. Funerals, memorial services, marriage ceremonies, and weddings would need to be regulated but supported through the various arms of the government to prevent the spread of Covid-19 at these functions. It cannot be argued that many families, health workers, and security personnel might need psychological support. This pandemic has caused so much anxiety and stress in a lot of people; social services need to facilitate education and awareness through availing hotlines which affected people can use to reach out, with provisions in place to protect their identity and dignity.

## Competing interests

The authors declare no competing interests.
